# Two CMOS Wilkinson Power Dividers Using High Slow-Wave and Low-Loss Transmission Lines

**DOI:** 10.3390/mi15081009

**Published:** 2024-08-05

**Authors:** Chatrpol Pakasiri, Wei-Sen Teng, Sen Wang

**Affiliations:** 1 College of Advanced Manufacturing Innovation, King Mongkut’s Institute of Technology Ladkrabang, Bangkok 10520, Thailand; chatrpol.pa@kmitl.ac.th; 2 Department of Electronic Engineering, National Taipei University of Technology, Taipei 10608, Taiwan; t108368047@ntut.org.tw

**Keywords:** Wilkinson power divider, transmission line, CMOS, coplanar waveguide (CPW), slow-wave factor (SWF), meandered line, low loss

## Abstract

This work presents two Wilkinson power dividers (WPDs) using multi-layer pseudo coplanar waveguide (PCPW) structures. The PCPW-based WPDs were designed, implemented, and verified in a standard 180 nm CMOS process. The proposed PCPW features high slow-wave and low-loss performances compared to other common transmission lines. The two WPDs are based on the same PCPW structure parameters in terms of line width, spacing, and used metal layers. One WPD was realized in a straight PCPW-based layout, and the other WPD was realized in a meandered PCPW-based layout. Both the two WPDs worked up to V-band frequencies, as expected, which also demonstrates that the PCPW guiding structure is less susceptible to the effects of meanderings on the propagation constant and characteristic impedance. The meandered design shows that the measured insertion losses were about 5.1 dB, and its return losses were better than 17.5 dB at 60 GHz. In addition, its isolation, amplitude imbalance, and phase imbalance were 18.5 dB, 0.03 dB, and 0.4°, respectively. The core area was merely 0.2 mm × 0.23 mm, or 1.8 × 10^−3^λ_o_^2^.

## 1. Introduction

**Figure 1:**
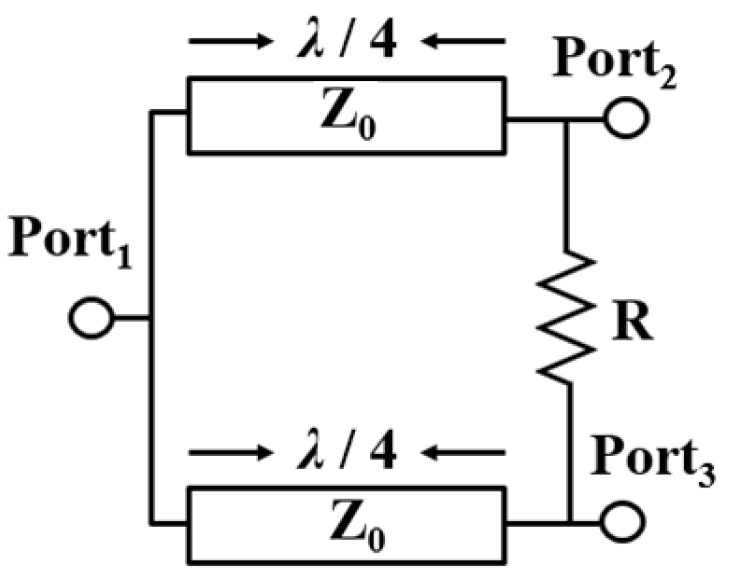
A Conventional WPD topology using two 70.7-Ω quarter-wavelength transmission lines and one 100-Ω isolation resistor.

Power dividers play an important role in the design of microwave circuits, which are widely used in power amplifiers, mixers, and phase shifters. The most widely used power divider is the Wilkinson power divider (WPD), using two 70.7-Ω quarter-wavelength transmission lines and one 100-Ω isolation resistor, as shown in Figure 1, which also offers equal power splitting with in-phase responses at output ports [1,2]. In addition, when the output port of the Wilkinson power divider is matched, the entire network will be lossless. WPDs also have advantages such as easy implementation and good port-to-port isolation. To date, there are many different transmission line (TL)-based or distributed-on-chip WPDs such as coupled lines [3], thin-film microstrip line (TFMSL) [4], coplanar waveguide (CPW) [5], and conductor-backed coplanar waveguide (CBCPW) [6], used to achieve acceptable loss or moderate chip size. Moreover, a CPW-like TL using the multi-layer CMOS process was also presented to achieve low loss at the Ka-band [7]. However, these TL-based designs are still area-consuming compared to other lumped designs [8,9,10,11].

The lumped WPDs formed by inductors and a capacitor were proposed to further replace the TLs, thus reducing the chip size significantly [8,9]. But the WPDs have a narrow bandwidth and poor insertion losses. Both the divider using inductive and capacitive loading [10] and the divider using transformers [11] feature low loss and compact size. However, they also suffered from poor isolation and amplitude/phase imbalances due to their lossy lumped components at millimeter-wave frequencies. The divider using stub-loaded elevated coplanar waveguide (ECPW) improved amplitude and phase imbalances [12]. However, the additional loaded elements also degrade its loss characteristic and increased chip areas. The divider with a loaded/modified CPW structure achieves extremely low loss because it is implemented on the glass-integrated passive device (GIPD) process, which offers low-low glass substrate with superior passive components such as inductors, capacitors, and TLs [13]. For the wideband on-chip divider, LC sections [14], cascaded lumped transmission lines [15]. have been used in increasing the bandwidth Once the power combiners or dividers are practical, they can be applied to many power systems [16].

Although the CMOS lumped power dividers have a compact chip area, they reveal poor agreement between simulation and measurement at high frequencies, especially at millimeter-wave frequencies due to the parasitics resulted from on-chip inductors. Moreover, the CMOS lossy substrate results in low-quality (*Q*) factor inductor and thus degrades the insertion of WPDs. In this paper, a TL-based multi-layer guiding structure called pseudo coplanar waveguide (PCPW) was incorporated into WPD designs. The multi-layer PCPW provides low-loss and high slow-wave performances. Then, its insertion losses and chip size are comparable with conventional TL-based designs and lumped designs, respectively. The remainder of this paper is organized as follows. Section 2 describes these conventional TLs and the design and analysis of the PCPW. Section 3 details the implementation and measurement of two PCPW-based WPDs. Finally, Section 4 concludes this paper.

## 2. Design and Analysis

### 2.1. Process Description and Conventional Transmission Lines

**Figure 2:**
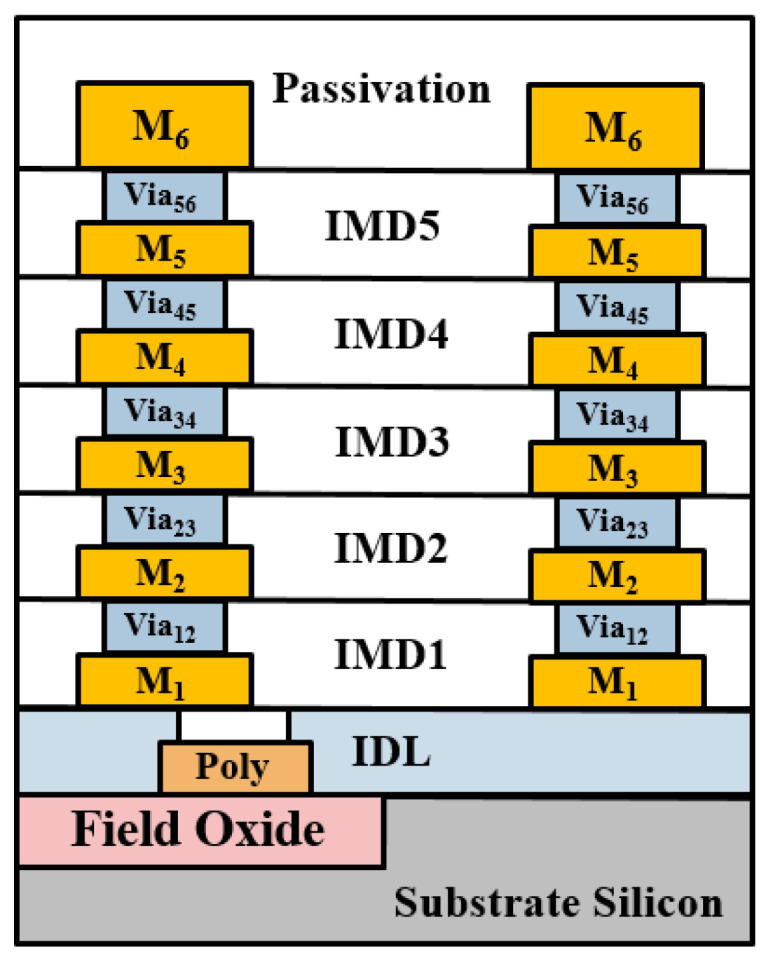
Cross-section of a standard 0.18 μm CMOS process.

**Figure 3:**
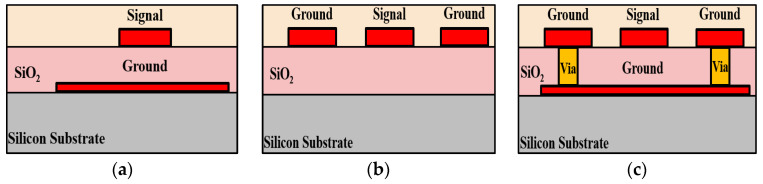
Cross-section view of (**a**) the thin-film microstrip line, (**b**) coplanar waveguide, and (**c**) conductor-back coplanar waveguide.

A standard 0.18 μm CMOS process offers one poly layer and six metal layers, as shown in Figure 2. The thickness of the top metal layer (M_6_) is 2.34 μm, and the thickness of other metal layers (M_1~5_) are all 0.53 μm. Typically, the M_1_ layer is also known as the bottom metal layer. Each layer of metal can be connected by Via layers. Except for the thickness of Via56, which is 1 μm, the thicknesses of the other Via layers are 0.85 μm. The poly layer is for the transistor’s gate and thus is above the substrate. The M_1_ and poly layers are separated by a 0.75 μm thick SiO_2_. The dielectric constant of the passivation layer and IMD_1-5_ layers are about 7.5 and 4, respectively. Due to the multi-layer technology, the CMOS process has a high degree of flexibility in designing different transmission lines. The most widely used transmission lines include thin-film microstrip lines (TFMSL) [17], coplanar waveguides (CPW) [18], and conductor-backed coplanar waveguides (CBCPW) [19]. Typically, thin-film microstrip lines (TFMSL) adopt the thick top metal layer (M_6_) as a signal trance for minimize conductor losses, and they use one of the other metal layers as a ground plane, as shown in Figure 3a. The biggest advantage of TFMSL is that it is suitable for circuit miniaturization. However, compared to other traditional transmission lines, it cannot withstand higher power transmission and has higher loss [20].

Figure 3b depicts the structure of the coplanar waveguide (CPW). The CPW is to place a signal trace at the center of two ground planes, so that two tiny narrow gaps are formed between the metal strip line and the ground planes [18]. The CPW is featured as its single metal formation, and the advantage of CPW is that the metal signal line in the middle is very close to the ground planes on both sides, so CPW is particularly easy to use when designing active circuits. In addition, the electrical characteristics of CPW are less affected by the thickness of the dielectrics. The characteristic impedance is determined by the width of the two sets of narrow gaps and the width of the metal strip line in the middle. Therefore, the achievable range of the characteristic impedance is relatively large compared to the TFMSL. The conductor-backed coplanar waveguide (CBCPW) is a derivative structure of the coplanar waveguide [19]. The difference is that CBCPW adds a ground plane on the substrate, as shown in Figure 3c. In addition to maintaining the advantages of CPW, CBCPW has a third ground conductor, so it has a better heat dissipation effect. However, because of this architecture, it is not easy to implement a transmission line with high characteristic impedance.

### 2.2. The Proposed Pseudo Coplanar Waveguide

**Figure 4:**
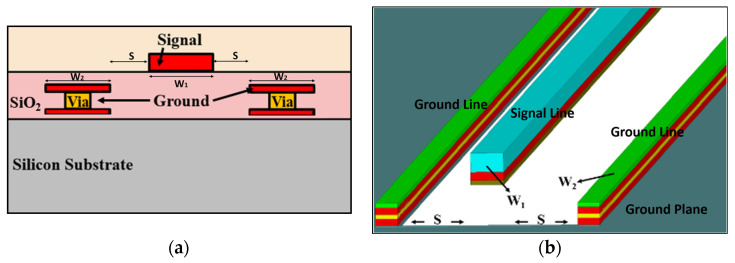
(**a**) Cross-sectional view and (**b**) 3D structure of the PCPW.

According to the above discussion, no matter how advanced the process is, the transmission line has become an irreplaceable key point. However, with the development of the times, there are now higher requirements for transmission lines, not only to pursue low loss, but also to achieve the effect of reducing the area. In this paper, the presented WPDs were designed by a novel transmission line, the so-called pseudo coplanar waveguide (PCPW) guiding structure. Figure 4a depicts the cross-sectional view of the PCPW structure. The main difference between the pseudo coplanar waveguide and the conventional coplanar waveguide (CPW) is that the signal line and ground plane are implemented on different metal layers, and the difference between the PCPW and CPW-like TLs in Ref. [7] is that a CPW-like TL is kept underneath the bottom metal ground plane, namely, its structure is also like CBCPW. Moreover, both the signal line and ground plane can be further realized by multi-layers, as shown in Figure 4b. The relative dielectric constants of intermedia-dielectric layers (SiO_2_) are about 4, and metal layers are made of AlCu, whose conductivity is about 2.8 × 10^7^ S/m. In this work, the signal line was composed of the top metal layer (M_6_) and the bottom layer (M_5_), which are connected by via_56_. The reference ground plane comprises the bottom layer (M_1_) and the two above layers (M_2_ and M_3_). Instead of a single layered signal line and ground plane, the multi-layered signal line and ground plane will decrease conductor losses due to their thick metal layers. And the equivalent capacitance between signal and ground will be higher, which also achieves a higher SWF than a single-layer ground plane. Moreover, the spacing (*S*) between the signal line and ground plane can also be tuned to lower equivalently parasitic capacitance, thus achieving high characteristic impedance, as shown in Figure 4b. The equivalent model of these TLs can be the lumped-element RLCG model, for which each element represents resistive loss, parasitic inductance, and capacitance.

Figure 4b also shows the structural parameters of PCPW, including the width of the signal line (*W*_1_), the width of the reference ground (*W*_2_), and the distance between the signal line and reference ground (*S*). Therefore, the PCPW features a high degree of design flexibility. Through these parameters, the characteristic impedance and electrical characteristics of PCPW can be determined, such as slow-wave factor (SWF), attenuation (α), and propagation constant (β). With the above variables, transmission lines with various characteristic impedances can be easily designed, so that they can be applied to various active and passive microwave circuits.

### 2.3. Comparison of Different Transmission Lines on CMOS

**Figure 5:**
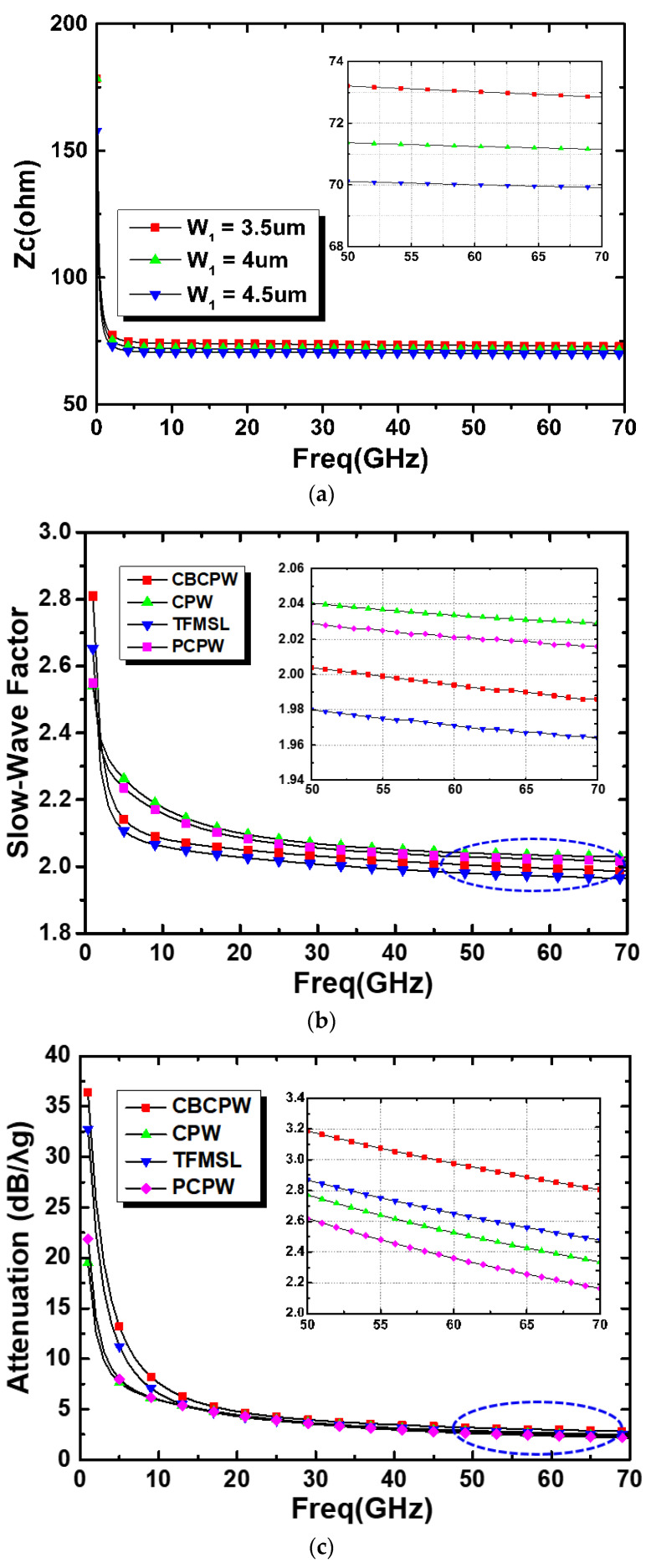
(**a**) Simulated *Z_c_* of the PCPW structure. Simulated (**b**) slow-wave factor (SWF) and (**c**) attenuation results of 70.7-Ω CBCPW, CPW, TFMSL, and PCPW structures.

The following simulations are evaluated by the full-wave EM simulator HFSS (high frequency structure simulator). In the simulation setup, the lumped port is assigned to each terminal of each TL, and the option of solve inside is also set to estimate the conductor losses correctly. The characteristic impedance of PCPW is dominated by signal linewidth (*W*_1_), and therefore, other structure parameters such as *S* and *W*_2_ can be fixed first. To obtain a 70.7-Ω PCPW for a power design, the parameters *S* and *W*_2_ are fixed to 10 μm and 3 μm, respectively. Then, the widths of signal are tuned at 3.5 μm, 4 μm, and 4.5 μm for optimum performance. Typically, the wide width of signal line results in high capacitance, and then a low characteristic impedance is obtained. Therefore, the trend of curves in Figure 5a is correct. Among these choices, the 4 μm wide signal line of PCPW is the closest to the characteristic impedance of 70.7 Ω. Figure 5a shows the simulated characteristic impedance (*Z*_c_) of the PCPW. Other commonly used 70.7-Ω transmission lines such as the thin-film microstrip line (TFMSL), coplanar waveguide (CPW), and conductor-backed coplanar waveguide (CBCPW) are also included in the figure for comparison in Figure 5b,c. The propagation constant (γ) and characteristic impedance (*Z*_c_) of these TLs can be derived from Equations (1) and (2), respectively [21,22].

(1) $$e^{\gamma L}=\frac{1-S_{11}^{2}+S_{21}^{2}+\sqrt{{\left(1+S_{11}^{2}-S_{21}^{2}\right)}^{2}-{\left(2S_{11}\right)}^{2}}}{2S_{11}}$$

(2) $$Z_{C}=Z_{O}\sqrt{\frac{{\left(1+S_{11}\right)}^{2}-S_{21}^{2}}{{\left(1-S_{11}\right)}^{2}-S_{21}^{2}}}$$

where the complex propagation constant is *γ* = *α* + *β*. The *β* is the phase constant, and α is the attenuation constant. The characteristic impedance of the line *Z_o_* is typically equal to 50 Ω. The physical length of the TL is denoted by L. Moreover, a slow-wave factor can be defined in Equation (3) as the ratio of the guiding wavelength (*λ_g_*) and the wavelength of free space (*λ_o_*) so that an electrical length of a TL can be estimated.

(3) $$SWF=\frac{\beta_{g}}{\beta_{o}}=\frac{\lambda_{o}}{\lambda_{g}}=\sqrt{\varepsilon_{r}\mu_{r}}≅\sqrt{\varepsilon_{r}}$$

Figure 5b shows the simulated *SWF* of each transmission line under the same characteristic impedance (70.7-Ohm). At 60 GHz, the slow-wave factor of CBCPW, CPW, TFMSL, and PCPW are 1.99, 2.03, 1.97, and 2.02, respectively. It can be found that the PCPW is similar to CPW in all transmission lines. On the other hand, the losses (dB/*λ_g_*) of CBCPW, CPW, TFMSL, and PCPW at 60 GHz are 2.99, 2.55, 2.67, and 2.38, respectively. Theoretically, the quality (*Q*) factor of TLs is proportional to the square root of frequencies, and therefore each TL should have lower attenuation or loss (dB/*λ_g_*) at higher frequencies. This can also be observed in Figure 5c. In summary, the simulated results show that the SWF of PCPW (2.02) is comparable to that of CBCPW (2.03), and the attenuation or loss of the PCPW is the lowest among these TLs. Therefore, the proposed multi-layered PCPW feature high slow-wave and low-loss characteristics are shown. The effects of meanderings on the propagation constant and characteristic impedance were also discussed in Ref. [21] by simulating many structural parameters to validity its feasibility. The key process variation parameter is the dielectric constant. Therefore, the dielectric constant within +/−10% variation is also conducted to observe its differences, and the variation only slightly results in frequency deviation. Then, with the 70.7-Ohm TLs, the last step is to optimize the isolation of WPDs by sweeping the resistor values because it is easier to adjust the values than the EM-simulated TLs.

## 3. Implementation and Experimental Results

**Figure 6:**
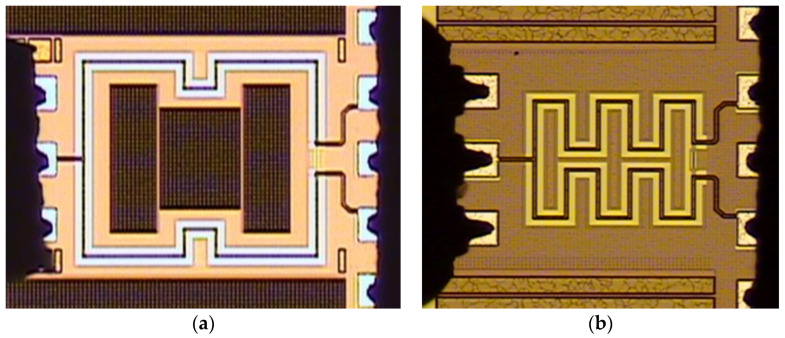
Chip photo of the two proposed WPDs. (**a**) Straight PCPW-based layout and (**b**) meandered PCPW-based layout.

There were two multi-layered PCPW Wilkinson power divider designs used in this paper for further comparison. The two WPDs were based on the same PCPW structure parameters in terms of line width, spacing, and used metal layers. One was straight PCPW-based WPD, and the other was meandered PCPW-based WPD. Both the two 60 GHz WPDs were designed and fabricated in a standard mixed-signal/RF bulk 0.18 μm CMOS process provided by the Taiwan Semiconductor Manufacturing Company (TSMC), Hsinchu, Taiwan The process provides a single poly layer and six metal layers (1P6M) for interconnections. The relative dielectric constants of intermedia-dielectric layers (SiO_2_) and silicon substrate were about 4 and 11.9, respectively. Moreover, the top metal layer (M_6_) had a thickness of 2.34 μm, and the other metal layers (M_1_~M_5_) were 0.53-μm. All metal layers were made of AlCu. Figure 6 shows the chip photo of the two WPDs using straight- and meandered-layout PCPW structures. The chip sizes excluding all testing pads of the straight-layout and meandered-layout WPDs were 0.625 mm × 0.6 mm and 0.2 mm × 0.23 mm, respectively. Both layouts were symmetrical, and there were some dummy metal fills placed inside and outside the two layouts to meet the design rules required by the foundry for metal planarization, as shown in Figure 6.

On-wafer measurements with the network analyzer (Keysight N5247A) were conducted to characterize the *S*-parameters of the two WPDs from 10 MHz to 67 GHz. All measured results were calibrated to eliminate the undesired parasitics of the testing pads. One ground–signal–ground Cascade^TM^ (GSG) RF probe was used on the left-hand side and one Cascade^TM^ GSGSG RF probe was used on the right-hand side, as shown in Figure 6. The two prototype designs were tested for more than three samples. For the measured insertion losses (|*S*_21_| or |*S*_31_|) and the measured center frequency of the designs, the sample-to-sample variations were less than 0.1 dB and 0.4 GHz, respectively. The following experimental results show the average data among the measured samples.

**Figure 7:**
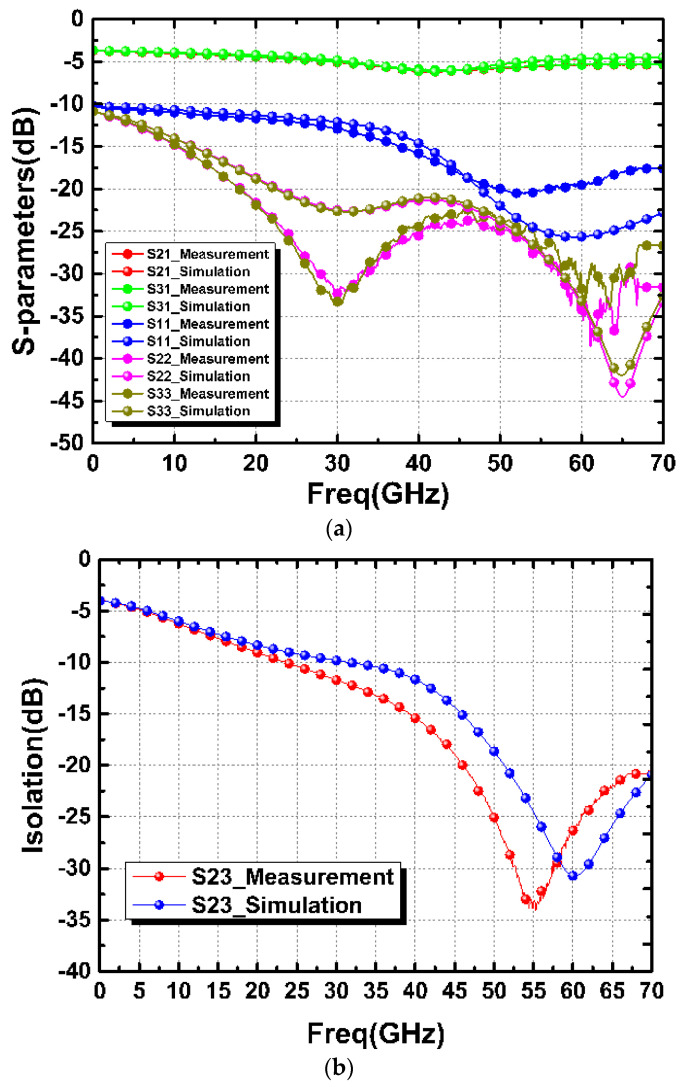

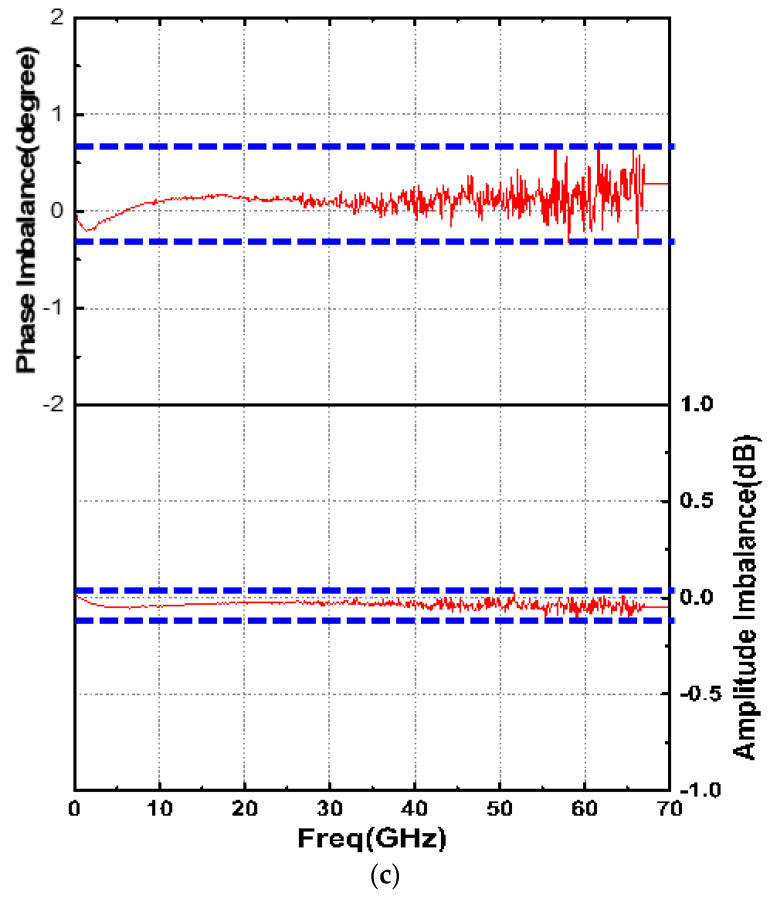
Simulated and measured results: (**a**) insertion and return losses, (**b**) isolation, and (**c**) phase and amplitude imbalances of the straight PCPW-based WPD.

**Figure 8:**
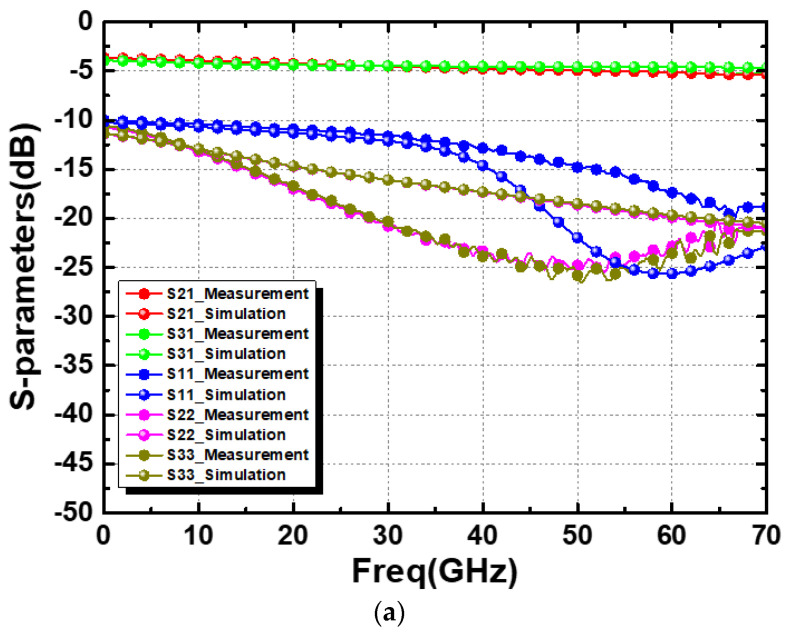

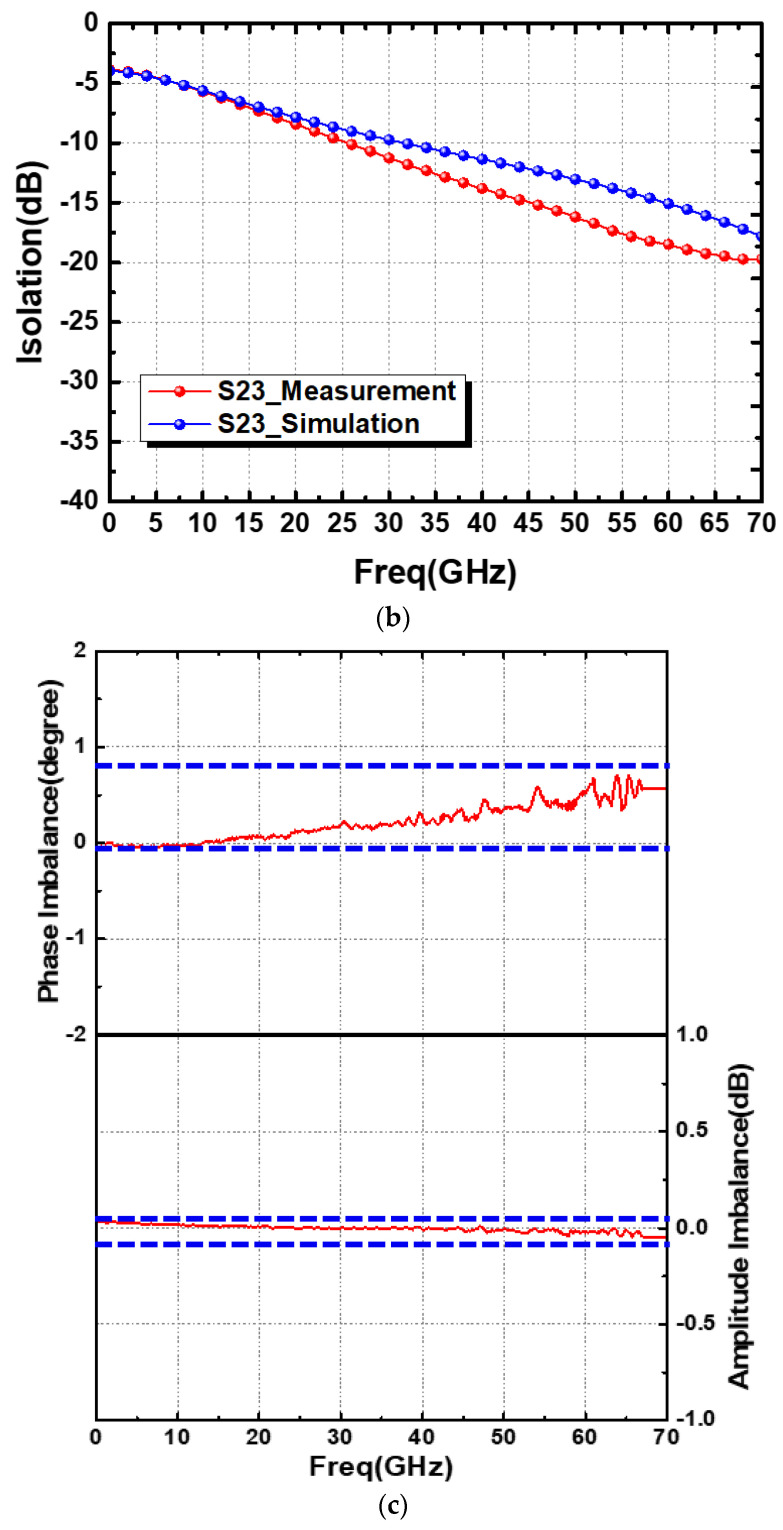
Simulated and measured results: (**a**) insertion and return losses, (**b**) isolation, and (**c**) phase and amplitude imbalances of the meandered PCPW-based WPD.

Figure 7 shows the simulated and measured results of the straight-based PCPW WPD. The measured |*S*_21_|, |*S*_31_|, |*S*_11_|, |*S*_22_|, and |*S*_33_| at 60 GHz were 5.39 dB, 5.32 dB, −19.56 dB, −31.6 dB, and −34.3 dB, respectively, as shown in Figure 7a. The measured |*S*_23_| (isolation) was 26.3 dB. Furthermore, the amplitude and phase imbalance were calculated from Figure 7a to be less than 0.1 dB and 0.7°, respectively. Similarly, the meandered design demonstrated that the measured |*S*_21_| and |*S*_31_| were about 5.1 dB, as shown in Figure 8a. All of the return losses were better than 17.5 dB at 60 GHz. Moreover, the measure isolation, amplitude imbalance, and phase imbalance were 18.5 dB, 0.03 dB, and 0.4°, respectively, as shown in Figure 8b,c.

In general, good agreements between simulated and measured results were observed, which shows the feasibility of the straight and meandered PCPW guiding structures. Both the two WPDs worked up to V-band frequencies, as expected, which also demonstrates that the PCPW guiding structure was less susceptible to the effects of meanderings on the propagation constant and characteristic impedance. The meandered design features a compact chip size, and therefore achieved better insertion loss, lower amplitude/phase imbalances, and poor isolation compared to the straight PCPW-based layout one. Finally, Table 1 summarizes the state-of-the-art on-chip WPDs. The used multilayered signal line and ground plane design decreased conductor losses, and they also achieved a higher SWF than a single-layer ground plane. Therefore, the proposed meandered WPD features excellent performances in isolation, amplitude, and phase imbalance, while it maintains a compact size and acceptable insertion loss. Moreover, the insertion losses and chip size of the meandered design are comparable with conventional TL-based designs and lumped designs, respectively.

## 4. Conclusions

Two V-band Wilkinson power dividers based on multi-layer PCPW were designed and implemented in a CMOS process. One WPD was realized in a straight PCPW-based layout, and the other WPD was realized in a meandered PCPW-based layout. Good agreements between simulated and measured results were observed in both designs, which demonstrates the feasibility of the straight and meandered PCPW guiding structures. The measured results also showed that both of the proposed WPDs were superior in terms of isolation, amplitude/phase imbalance, and compact chip size among some works in literature. The proposed designs are well suited for popular power combining and splitting in high-ouput power and high-linearity power amplifier designs, as well as for the 60 GHz wireless communication systems.

## Figures and Tables

**Table 1:** Comparison of previously reported on-chip WPDs.

Ref.	[10]	[11]	[12]	[13]	This Work#1	This Work#2
Process	180 nm CMOS	130 nm SiGe BiCMOS	90 nm CMOS	GIPD	180 nm CMOS	
Topology	LC loading	Transformer	Stub-loaded ECPW	Loaded/modified CPW	Straight PCPW	Meandered PCPW
Frequency (GHz)	67	62	67	67	60	
*I.L.* (dB)	<5	4.4	5.4	<3.8	5.4	5.1
Isolation (dB)	15	15	14	>10	26.3	18.5
Amplitude imbalance (dB)	<0.45	<0.2	0.16	N/A	<0.1	<0.03
Phase imbalance (°)	<2	<5	0.45	<0.3	<0.7	<0.4
Area (λ_0_^2^)	0.0021	0.0012	0.0034	0.0877	0.015	0.0018

## Data Availability

The original contributions presented in the study are included in the article, further inquiries can be directed to the corresponding author.
